# Drivers and Barriers to Acceptance of Web-Based Aftercare of Patients in Inpatient Routine Care: A Cross-Sectional Survey

**DOI:** 10.2196/jmir.6003

**Published:** 2016-12-23

**Authors:** Severin Hennemann, Manfred E Beutel, Rüdiger Zwerenz

**Affiliations:** ^1^ Department of Psychosomatic Medicine and Psychotherapy University Medical Center Gutenberg University Mainz Mainz Germany; ^2^ Department of Clinical Psychology, Psychotherapy and Experimental Psychopathology Institute of Psychology University of Mainz Mainz Germany

**Keywords:** eHealth, attitude to health, aftercare, rehabilitation, survey

## Abstract

**Background:**

Web-based aftercare can help to stabilize treatment effects and support transition after inpatient treatment, yet uptake by patients seems limited in routine care and little is known about the mechanisms of adoption and implementation.

**Objective:**

The aim of this study was to (1) determine acceptance of Web-based aftercare and (2) explore its drivers and barriers in different subgroups of a mixed inpatient sample.

**Method:**

In a cross-sectional design, 38.3% (374/977) of the inpatients from a broad spectrum of diagnostic groups (psychosomatic, cardiologic, orthopedic, pediatric, and substance-related disorders) filled out a self-administered questionnaire prior to discharge. Drivers and barriers to patients’ acceptance of Web-based aftercare were examined based on an extension to the “unified theory of acceptance and use of technology” (UTAUT). In total, 16.7% (59/353) of the participants indicated prior use of eHealth interventions.

**Results:**

Acceptance (min 1, max 5) was low (mean 2.56, SD 1.22) and differed between diagnostic groups (Welch *F*_4,133.10_ =7.77, *P*<.001), with highest acceptance in adolescent patients (mean 3.46, SD 1.42). Acceptance was significantly predicted by 3 UTAUT predictors: social influence (beta=.39, *P*<.001), performance expectancy (beta=.31, *P*<.001), and effort expectancy (beta=.22, *P*<.001). Furthermore, stress due to permanent availability (beta=−.09, *P*=.01) was negatively associated with acceptance.

**Conclusion:**

This study demonstrated a limited acceptance of Web-based aftercare in inpatients. Expectations, social environment’s attitude, and negative experience with permanent availability influence eHealth acceptance. Improving implementation, therefore, means increasing eHealth experience and literacy and facilitating positive attitudes in patients and health professionals through education and reduction of misconceptions about effectiveness or usability.

## Introduction

### Background

The digitalization of health care brought forth a broad range of effective eHealth interventions for various somatic [1] and mental health problems [2]. The effects of guided interventions for some mental disorders turned out to be comparable to those of traditional face-to-face therapy [3]. eHealth interventions can be a cost-efficient, time-flexible, and location-independent extension of existing health care [4], particularly in areas such as prevention, as aftercare or blended care. In the case of inpatient rehabilitation, previous research focused on Web-based aftercare [5], as the transition to daily life after inpatient treatment is a critical phase, with elevated risk of relapse, sick leave, return to adverse health behavior, or even inpatient readmission [6]. Therefore, clinics often recommend participation in outpatient treatment and aftercare (eg, physiotherapy, vocational rehabilitation, educative group sessions). However, organizational constraints or limited accessibility, concerns about anonymity, or negative treatment expectancies can reduce the utilization of conventional outpatient treatment or aftercare [7,8]. In this regard, Köpke [9] describes insufficient uptake rates of conventional aftercare interventions between 18.9% for somatic and 69.6% for psychosomatic disorders after inpatient rehabilitation in Germany.

Previous Web-based aftercare interventions have been developed and evaluated particularly for psychosomatic inpatients, for example, cognitive behavioral therapy (CBT)–based Internet platforms [10,11] or mobile interventions [12], providing modular or consecutive treatment elements with a combination of health-related information and interactive tasks to promote health behavior (see also Multimedia Appendix 1). Furthermore, interventions differ by amount of guidance by health experts [13], who support health behavior and self-efficacy through synchronous (eg, video- or chat-based) [14] or asynchronous (email, short message service) [11] interaction and task-related feedback. Most Web-based aftercare interventions with psychosomatic focus are CBT based, whereas more recent developments include psychodynamic [15] or acceptance- and commitment-based approaches [16].

However, despite an increased proportion of inpatients with pronounced work-related stress and subsequently an elevated risk of early retirement [17], occupational aspects play a subsidiary role in eHealth interventions in the context of inpatient treatment. Only a few projects explicitly addressed work-related stress in clinical or subclinical samples and yielded mixed effects [15,18,19]. As the digitalization of health care is still in its early stages in Germany, eHealth interventions are scarce in inpatient care, with only 9% of studies on eHealth interventions in the area of rehabilitation according to Eichenberg and Ott [20].

A fundamental precondition to the implementation of eHealth interventions is the willingness to utilize and adopt new technologies by patients and help seekers [21]. Despite their efficacy, uptake of and adherence in eHealth interventions pose a challenge for implementation in routine care [22], and mechanisms of uptake of eHealth interventions and their relevance to implementation have not been studied sufficiently yet.

### Acceptance of E-Mental Health Interventions

Acceptance can imply the utilization of an intervention, as expressed in uptake rates, adherence, or satisfaction. Following theoretical models of acceptance of information technology, acceptance can also be defined as the behavioral intention to use eHealth interventions [23,24] as an essential prerequisite to adoption and implementation of eHealth interventions.

Based on their review of 36 studies, Waller and Gilbody [25] concluded that participants’ satisfaction with computerized CBT (cCBT) is usually high; however, the willingness to participate is limited, with only 38% starting the intervention after recruitment. Other findings are inconsistent, with various studies documenting a low uptake rate of e-mental health interventions by patients [21,25] or limited acceptance in the general population [26,27].

According to a recent representative population survey in Germany, less than 10% of the interviewed participants could imagine using psychological Web-based support and merely 2% had already used such services in the past [27]. Whereas recent studies point to the acceptance of eHealth records and computer-based diagnostic systems in inpatient treatment [28,29], preliminary evidence on some Web-based aftercare projects in German rehabilitation shows fluctuating uptake rates between 21% and 62% [30,12]. Multimedia Appendix 1 compares acceptance ratings (eg, uptake, adherence) of exemplary Web-based aftercare interventions including guided self-help [11], blended [31], mobile-based [12,32], and chat- or education based [33-36] interventions. The results of Pfaudler et al [33] and Küffner [35] indicate organizational and technical problems as pivotal barriers to adoption by inpatients.

### Methodical Shortcomings of Acceptance Assessment

Although acceptance ratings have been included in most studies on eHealth interventions by now, methodical shortcomings may somehow limit their validity. Since acceptance is typically assessed retrospectively, no information about genuine attitudes toward this form of health promotion or reasons for intention to use an intervention is provided. Furthermore, acceptance ratings may be confounded with intervention satisfaction and do not reflect eHealth acceptance or attitudes. A selective dropout of dissatisfied participants and participation of Internet-oriented patients may also positively bias acceptance ratings. The lack of sufficient theoretical foundation and valid or consistent instruments of acceptance evaluation can further be criticized [21]. A recent example for a methodical valuable approach is the 4-dimensional “Attitudes toward psychological online interventions” questionnaire (APOI), which has been cross-validated in a large sample of individuals with symptoms of depression [37]. Furthermore, previous studies focused on acceptance in rather selected patient samples. Hence, comprehensive findings from inpatient treatment are scarce. Altogether, these methodological shortcomings may illustrate the importance of a differentiated, theory-based, and transdiagnostic assessment of eHealth acceptance and its preconditions.

### Barriers and Resources to eHealth Acceptance

Research on predictors of eHealth acceptance has established the technology acceptance model (TAM) [23], and its advancement, the unified theory of acceptance and use of technology (UTAUT) [24] in several studies in patient populations [38-41] or health professionals [42-44]. Both models integrate traditional motivational theories such as the theory of planned behavior [45] or Bandura’s social cognitive theory [46]. Since the UTAUT is more differentiated regarding predictors on acceptance and has recently been used in similar research questions [38-41], we adapted this model to the following study. The UTAUT postulates 4 core predictors of behavioral intention: performance expectancy (expected benefit of technology use), effort expectancy (expected ease of use), social influence (attitude of significant others toward using the technology), and facilitating conditions (organizational or technical resources and preconditions necessary to technology use). In previous studies, performance expectancy proved to be the most important predictor of eHealth acceptance [44,47,48]. In a meta-analysis of 37 studies testing the UTAUT in various contexts, Taiwo and Downe [48] found a medium aggregated effect size of correlation with acceptance (z-transformed r=.54) and somewhat smaller effect sizes for the remaining model predictors (.38-.44).

However, research also points out the complex nature of acceptance and its determinants [42,43]. Therefore, the predictive model of eHealth acceptance needs to be extended and adapted to the context of different target groups. Typically, sociodemographic characteristics such as younger age, higher educational level, Internet access, and technical experience have been associated with acceptance of eHealth interventions in several studies [2,27,49,50]. Further potential barriers to acceptance include Internet anxiety [40,51], low Internet orientation in health problems [27], insufficient knowledge of eHealth interventions [27,52], rural residence [53], or reservations regarding data security or impersonal interaction [54,55]. However, barriers or facilitators to eHealth adoption in inpatient routine care have not been studied sufficiently yet.

### Goals of This Study

This study aimed to (1) determine the current status of acceptance of Web-based aftercare with a focus on work-related stress and (2) explore its drivers and barriers based on an extended UTAUT model in a mixed sample of inpatients in a cross-sectional survey. Besides previously studied Internet-related predictors, we were interested in the influence of eHealth literacy, which is defined as the ability to find, evaluate, and utilize Internet-based health information to deal with health problems [56]. Furthermore, we aimed to extend existing literature on the role of self-efficacy [57] in the eHealth context. Since the evidence on the influence of symptom severity and uptake of e-mental health interventions is mixed [11,58], we aimed to explore the relation of mental and occupational distress with acceptance. Also, since modern technologies increase digital load and availability [59], we were interested in the impact of stress due to constant availability (email, social media) on acceptance.

We postulated positive relations between UTAUT- and Internet-related predictors as well as self-efficacy. We assumed that Internet anxiety and stress through permanent availability would be negatively associated with acceptance ratings. Furthermore, we investigated subgroup-specific differences. Here, we expected younger age, higher educational status, and urban residency to be associated with higher acceptance.

## Methods

### Study Design

A cross-sectional approach was adopted to compare acceptance and its predictors in different inpatient diagnostic groups with a self-administered questionnaire. The survey was based on a qualitative pilot study, in which inpatients of various diagnostic groups were interviewed on health-related Internet use and barriers or resources to eHealth utilization in semistructured interviews. The questionnaires were built upon previous findings on predictors of acceptance, which were adapted to this study. In a consensual approach, the content, clarity, and face validity of the items were appraised by internal and external researchers and pretested in one of the cooperation clinics. The survey was conducted in 4 inpatient rehabilitation centers of the German statutory pension insurance scheme, covering a broad range of the most common diagnostic groups: psychosomatic medicine and psycho-oncology (PSY), orthopedics (OPE), cardiology (CAR), pediatric disorders of adolescent patients (PED), and substance use disorders (SUD).

### Participants and Recruitment

Participants were recruited consecutively by 4 research assistants from August 2015 to January 2016. Participants were required to be aged 14 or above due to ethical rules. Inpatients with at least two weeks of treatment or half of treatment completed were included in the study. Since the focus on work-related stress in Web-based aftercare, retired patients were excluded from further analyses. Of 977 eligible patients who received a questionnaire, 374 (38.3%) responded. Participants were first informed at admission (eg, in an introductory course or at intake) and recruited subsequent to a regular group screening prior to discharge. After oral and written information, patients could fill out the paper-pencil questionnaire. The survey contained 69 questions (59 in pediatric sample, as items regarding occupational aspects were omitted) and took about 15 minutes to fill out. The survey was completely anonymous. No written consent was needed for participants older than 15 years. However, 14-year-old participants and their parents had to fill out a written consent. Gift cards served as an incentive for participation. Participants who wanted to take part in the draw of the gift cards could leave their contact details, which were detached from the questionnaire and kept separately and inaccessible to the project manager to ensure anonymity. All procedures involved in the study were approved by the ethics committee of the Federal State of Rhineland Palatinate, Germany (Ref. No. 9434-F) and the data protection commissioner of the German statutory pension insurance scheme.

### Measures

#### Primary Outcome: Acceptance of Web-Based Aftercare

Based on the UTAUT [23], acceptance was operationalized as the intention to use Web-based aftercare. Acceptance was measured using 3 items (Table 1). All items were rated on 5-point Likert scales ranging from (1) “totally disagree” to (5) “totally agree,” with higher scores indicating elevated acceptance. The items were adapted from previous studies [23,38-40,47]. Following a short introduction with general information about Web-based aftercare, the prefix to all items was “A Web-based aftercare for the management of work-related stress...” (for adolescent patients: “Aftercare delivered over the Internet...”). Reliability was calculated as Cronbach alpha. To avoid underestimation of true reliability, the internal consistency of 2-item subscales was not calculated [60]. Cronbach alpha of the acceptance scale in this study was .85.

**Table 1:** Adapted items of the UTAUT model and references of original studies.

Variable	Items
Behavioral Intention	“I would like to try a Web-based aftercare.”^a,b,c^
	“I would use a Web-based aftercare if offered to me.”^a,b,c^
	“A Web-based aftercare would be worth paying for.”^a,b,c^
Social influence	“People close to me would approve a Web-based aftercare.”^a,b,c,d^
	“My general practitioner would approve the use of a Web-based aftercare.”^a,b,c^
	“My friends would approve a Web-based aftercare.”^e^
Performance expectancy	“A Web-based aftercare could improve my work-related well-being.”^a,b,c^
	“A Web-based aftercare could help me with work-related stress.”^a,b,c^
	“A Web-based aftercare could help me to improve my personal health.”^a,b,c,e^
	“A Web-based aftercare could help me with my health problems.”^a,b,c,e^
Effort expectancy	“A Web-based aftercare would be easy to operate and comprehend.”^a,b,c,d,f^
	“I could arrange using a Web-based aftercare in my everyday life.”^g^
Facilitating conditions	“I have the necessary technical preconditions for using a Web-based aftercare.”^a,b,c,d^
	“I possess the required technical knowledge to utilize a Web-based aftercare.”^c,d,f^

^a^Baumeister et al [38].

^b^Baumeister et al [39].

^c^Ebert et al [40].

^d^Venkatesh et al [23].

^e^Items used in the adolescent sample (PED).

^f^Liu et al [47].

^g^Self-constructed.

#### Secondary Outcome: Predictors of Acceptance

The UTAUT predictors were measured using 2 items each, adapted from previous studies [23,38-40,47]. Answers were rated on a 5-point Likert scale ranging from from (1) “totally disagree” to (5) “totally agree.” The items of the UTAUT predictors used in the questionnaire and the original studies they were adapted from are listed in Table 1.

Further differential factors were included: *knowledge of eHealth interventions* was measured in 2 items following a short explanation of eHealth interventions based on a previous study by Ebert et al [40]. Two items measuring *Internet anxiety* were adapted from previous studies [38-40]. Answers were rated analogous on a 5-point Likert scale ranging from (1) “totally disagree” to (5) “totally agree.” The frequency (from [1] “never” to [5] “always”) of *health-related Internet and mobile use* was evaluated for different applications (fitness, vital parameters, nutrition, mental and occupational problems). *Self-efficacy* was assessed with the German general self-efficacy short form (ASKU) [61], consisting of 3 items with responses rated on a 5-point Likert-type scale ranging from (1) “does not apply at all” to (5) “applies completely.” The questionnaire has proven to be reliable with alpha=.81 to .86 [62]. Cronbach alpha in this study was .89.

*eHealth literacy* was measured with the German adaption of the eHealth literacy scale (eHEALS) [63], consisting of 8 items. Answers were rated on a 5-point Likert scale ranging from (1) “strongly disagree” to (5) “strongly agree.” Previous research by Norman et al [64] showed a high internal consistency of .88. For economic reasons, the questionnaire for adolescent patients included only 3 items of the eHEALS. Items were selected regarding linguistic appropriateness for the target population and high factorial loadings [63]. Cronbach alpha of the original scale in this study was .95.

*Mental distress* was assessed with the Patient Health Questionnaire-4 (PHQ-4) as a brief screening instrument for depression and anxiety [65] in the adult patient sample. In 4 items with answers on a 4-point scale ranging from 0 (“Not at all) to 3 (“Nearly every day”), the extent of symptoms in the last 2 weeks was evaluated. Sum scores can be categorized as normal (0-2), mild (3-5), moderate (6-8), and severe (9-12) [65]. Cronbach alpha in this study was .93.

**Figure 1:**
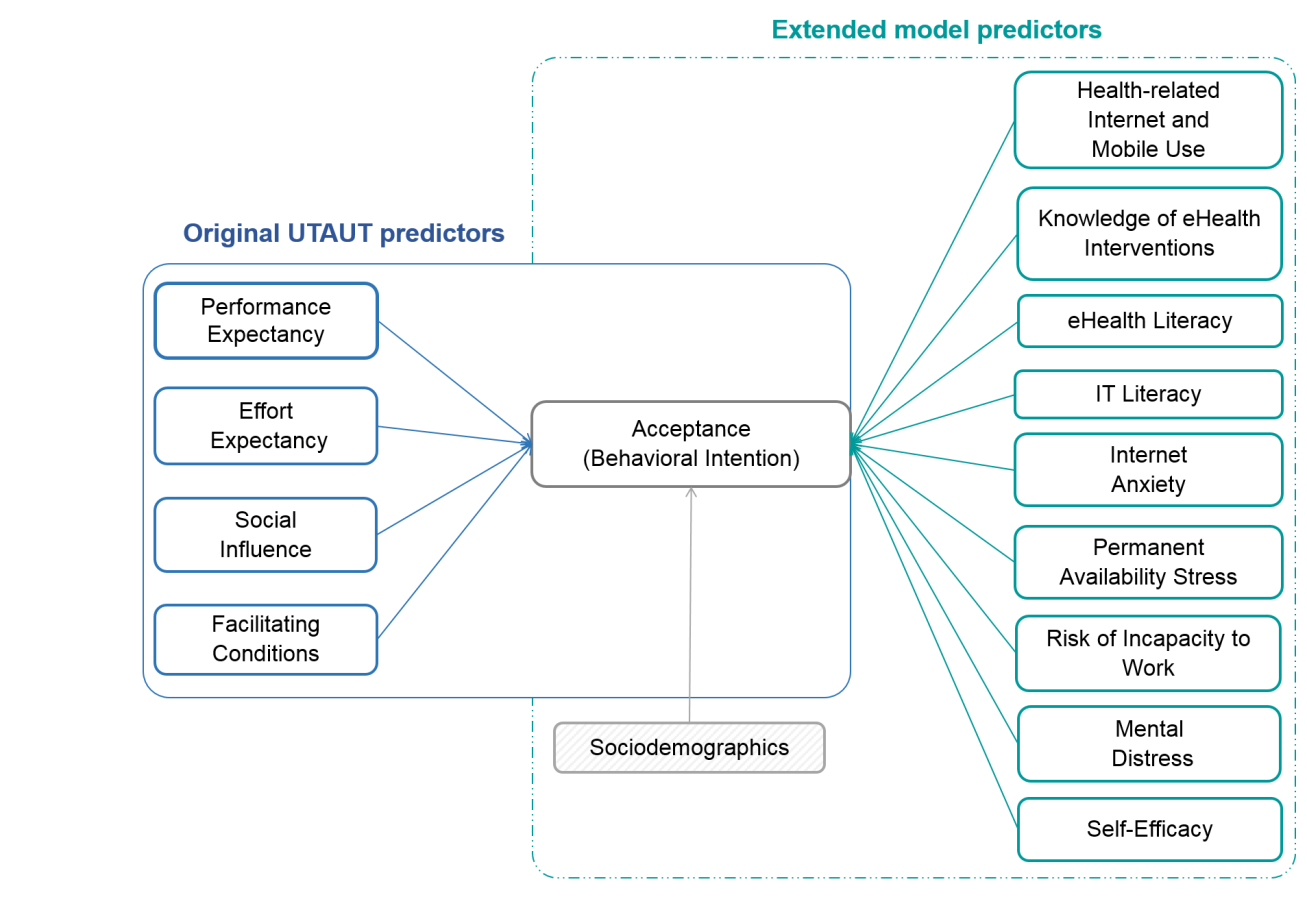
Research model based on the UTAUT (left) and extended predictors (right). UTAUT: unified theory of acceptance and use of technology.

Furthermore, we measured the *subjective risk of incapacity to work* with the 3-item subjective prognosis of work ability scale (SPE) [66] in adult participants. Answers were dichotomized, and sum scores ranged from 0 to 3. Probability of retirement doubles with each scale interval [67]. Cronbach alpha in this study was .70. Additionally, the influence of *IT-literacy* [answers ranging from (1) “very little knowledge” to (5) “very good knowledge”] and *stress through permanent availability* (level of agreement on a 5-point Likert-type scale from [1] “strongly disagree” to [5] “strongly agree”) on acceptance were examined in self-constructed items.

To further explore preconditions of intended usage, participants could state subjective advantages or disadvantages of using a Web-based aftercare. Finally, 1 item measured preferences for different forms of Internet-based support. The research model is depicted in Figure 1.

#### Sociodemographic Characteristics

The survey contained items regarding sex, age, education or current school type, population of residence, Internet access, time spent on Internet in free time per day, occupational status prior to rehabilitation, and days on sick leave (from [1] “less than 9 days” to [5] “100-365 days”) from the Work Ability Index [68]. One item differentiating between types of experience with eHealth interventions was adapted from a previous study by Eichenberg et al [27].

### Statistical Analyses

The data were analyzed using SPSS Statistics version 23 (IBM). Out of 374 datasets, 18 individuals with missing values exceeding 50% (mean 77.73% missing, SD 17.37) were excluded from further analysis [69]. Furthermore, 18 individuals who indicated being retired were excluded, resulting in 338 participants analyzed. We addressed missing data under the assumption of data missing at random with multiple imputation technique in SPSS including all analysis variables [62]. To demonstrate the extent of missing data, sociodemographic statistics (Multimedia Appendix 2) were calculated with observed data, whereas further analyses were based on imputed values (0.6%-15.7% missing values per variable). Imputed statistics were comparable to observed statistics. Group differences of demographics, primary, and secondary outcomes were analyzed using the chi-square test or G-test, analysis of variance with post-hoc comparisons (Games-Howell), or independent *t* tests. In case of variance inhomogeneity, Welch F-ratio was calculated. Acceptance scores were further categorized by mean to describe low (1-2.34), moderate (2.35-3.67), and high (3.68-5) acceptance, and frequency of categories was calculated. The predictive model of acceptance was tested using multiple hierarchical regression. Predictors were included blockwise: (1) sociodemographic and background variables (mental and occupational distress, self-efficacy), (2) Internet-related variables, and (3) UTAUT predictors. According to Midi, Sarkar, and Rana [70], no sign of severe multicollinearity could be detected and all predictors could enter the regression. To perform further between-group comparisons of acceptance, mental distress and risk of incapacity to work were dichotomized with a median split. Open answers regarding advantages and disadvantages of Web-based aftercare were rated according to qualitative content analysis [71] by 2 independent raters following a-priori consensually developed categories. An inter-rater reliability analysis using Cohen kappa statistic was performed to evaluate consistency.

## Results

### Sociodemographic Characteristics

General characteristics of 374 participants are shown in Multimedia Appendix 2. More men than women participated in the study, and mean age was 45.49 years (SD 14.17, min 14, max 78). The age range of the pediatric subsample was 14-18 years. The majority of adult inpatients indicated being employed prior to treatment. Educational status was moderate overall. A total of 59 (16.7%, 59/353) participants stated prior eHealth use. Significant differences between diagnostic groups were found in all sociodemographic variables with exception to the distribution of wearables and prior e-mental health usage, primarily in comparison to the pediatric subsample.

### Acceptance of Web-Based Aftercare

**Figure 2:**
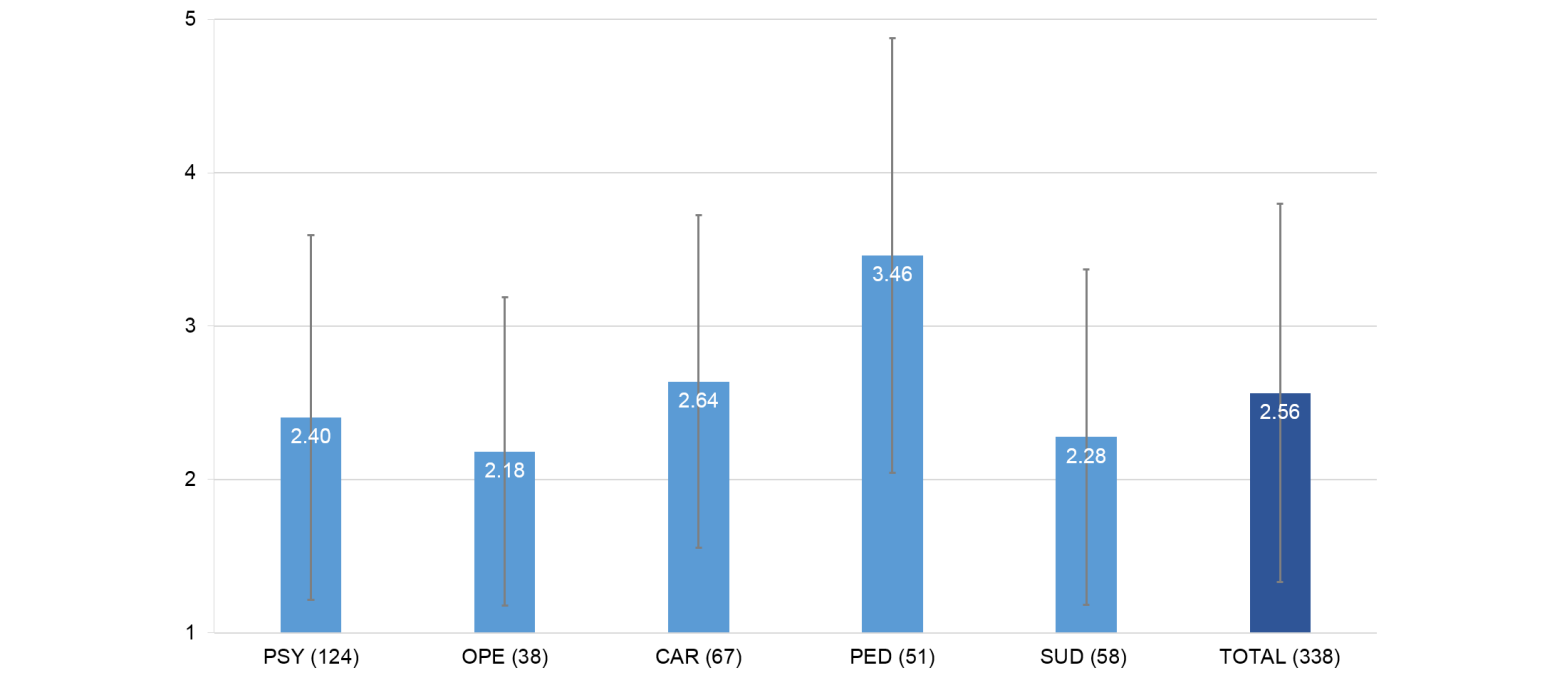
Acceptance (means) of Web-based aftercare between diagnostic groups. Sample size in brackets. Error bars represent standard deviations.

Acceptance of Web-based aftercare was overall low in patients with a mean of 2.56 (SD 1.23). The highest acceptance was found in pediatric patients with a mean of 3.46 (SD 1.42), the lowest in orthopedic patients with a mean of 2.18 (SD 1.00). The diagnostic groups differed statistically significantly (Welch *F*_4,133.10_=7.77, *P*<.001). Post-hoc comparisons demonstrated significant differences in all disorder groups compared with the pediatric patients, but not between other diagnostic groups. When including sociodemographic variables as covariates, diagnostic group differences remained marginally significant (*F*_4, 326_=2.41, *P*=.05). Acceptance between diagnostic groups is depicted in Figure 2.

Further analyses revealed that 48.8% (165/338) could be categorized with a low, 32.8% (111/338) with a moderate, and 18.4% (62/338) with a high acceptance. This pattern only differed in cardiological (42% [28/67] “low,” 48% [32/67] “moderate,” 10% [7/67] “high”) and pediatric patients (22% [11/51] “low,” 24% [12/51] “moderate,” 55% [28/51] “high”). Only 10.2% (31/305) participants with valid answers agreed that they would be willing to pay for participation in a Web-based aftercare.

Acceptance differed significantly between age groups (*F*_3,342_=9.97, *P*<.001) with highest acceptance in the youngest quartile (14-26 years). Post-hoc tests revealed significant differences between the youngest and all other age groups. Further data inspection of sociodemographic variables revealed no differences in acceptance regarding gender, population of residence, and occupational status. However, prior eHealth use, higher educational status, and private Internet access were associated with higher acceptance. Table 2 contains acceptance scores as a function of demographics.

**Table 2:** Differences in acceptance by demographics (N=338).

Variable		n	Mean (SD)	Test	*P* value
**Sex**				
	Male	198	2.62 (1.21)	*t*_336_=0.93	.35
	Female	140	2.49 (1.26)		
**Age in years**				
	14-26	57	3.34 (1.42)	*F*_3,334_=9.57	*P*<.001
	27-39	32	2.40 (1.18)		
	40-52	130	2.41 (1.11)		
	53-65	119	2.40 (1.23)		
**Educational status**				
	No graduation	10	1.95 (1.04)	*F*_2,335_=8.00	*P*<.001
	Secondary school	277	2.48 (1.18)		
	A-level	51	3.14 (1.38)		
**eHealth experience**				
	Yes	56	2.44 (1.20)	Mann-Whitney *U*=5315	*P*<.001
	No	282	3.16 (1.24)		
**Internet access**				
	Yes	296	2.65 (1.23)	*t*_336_=−3.60	*P*<.001
	No	42	1.98 (1.11)		
**Occupational status** (287)^a^				
	Employed	195	2.43 (1.13)	*F*_2,284_=0.41	.66
	Unemployed	44	2.26 (1.11)		
	Sick leave	48	2.42 (1.14)		
**Population of residence**				
	Rural	145	2.53 (1.27)	*t*_336_=−0.41	.68
	Urban	193	2.59 (1.21)		

^a^Retired and adolescent patients (PED) excluded.

### Drivers and Barriers to Acceptance

Multiple hierarchical regression in adult inpatients (PSY, OPE, CAR, SUD) showed that out of the demographic and background variables entered in the first step, gender (beta=–0.13, *P*=.04) and Internet access (beta=0.13, *P*=.03) predicted acceptance, although explained variance was low (*R*^2^=.06, *F*_9,272_=2.09, *P*=.03). When entering Internet-related variables, explained variance increased significantly (*R*^2^=.17, *F*_17,264_=3.22, *P*<.001), but only health-related Internet and mobile use significantly predicted acceptance (beta=0.22, *P*<.001). The full regressive model including UTAUT variables showed high explained variance (*R*^2^=.78, *F*_21,260_=43.86, *P*<.001). Social influence (beta=.39, *P*<.001), performance expectancy (beta=.31, *P*<.001), and effort expectancy (beta=.22 *, P*<.001) significantly predicted acceptance, whereas facilitating conditions did not reach significance (Table 3). Furthermore, stress through permanent availability (beta=–.09, *P*=.01) could be observed as a fourth significant predictor. Table 3 contains the regression parameters of the complete regression model.

In a separate regression analysis of the adolescent subsample (PED), explained variance of acceptance significantly improved in the model including UTAUT-predictors (*R²* = .85, *F*_18,32_=10.28, *P*<.001) in comparison to the previous steps (demographic variables only: *R²* = .24, *F*_6,44_=2.33, *P*=.05; demographic and Internet-related variables: *R²*=.43, *F*_14,36_=1.94, *P*=.06). However, of the UTAUT-predictors, only performance expectancy (beta=.67, *P*<.001) significantly predicted acceptance of Web-based aftercare. Knowledge of eHealth interventions (beta=.21, *P*=.02) and IT literacy (beta=−.22, *P*=.04) proved to be further predictors of acceptance in the full model. The correlation matrix of the total sample can be found in the Multimedia Appendix 3. Absolute values of intercorrelations ranged from |0.0| to |0.79|.

**Table 3:** Regression model of acceptance (full model, adult sample, N=282).

Predictor		b	Standard error	95% CI	Beta	*P* value
	Constant	.08	.44	−0.78 to 0.94		.86
**Sociodemographic predictors**						
	Sex	.03	.07	−0.11 to 0.18	.01	.64
	Age	.01	.00	0.00 to 0.02	.05	.17
	Days on sick leave	–.01	.03	−.0.06 to 0.05	−.01	.76
	Internet access	–.05	.11	−0.27 to 0.18	−.01	.68
	Population of residence	–.11	.07	−0.25 to 0.03	−.05	.12
	Educational status	–.06	.09	−0.24 to 0.11	−.02	.49
	Self-efficacy^a^	–.01	.05	−0.10 to 0.08	−.01	.80
	Mental distress^b^	.01	.01	−0.02 to 0.03	.02	.66
	Risk of incapacity to work^c^	–.01	.03	−0.07 to 0.06	−.01	.85
**Internet-related predictors**						
	eHealth literacy^d^	.01	.06	−0.10 to 0.12	.01	.90
	Internet anxiety	.02	.04	−0.06 to 0.10	.02	.67
	Knowledge of eHealth interventions	–.06	.04	−0.14 to 0.02	−.06	.14
	Time on Internet	–.01	.02	−0.04 to 0.03	−.01	.71
	eHealth experience	.18	.11	−0.03 to 0.39	.05	.09
	Health-related Internet and mobile use	.07	.06	−0.04 to 0.19	.04	.21
	IT literacy	.00	.04	−0.09 to 0.08	.00	.95
	Permanent availability stress	–.08	.03	−0.14 to −0.02	−.09	.01
**UTAUT-predictors**						
	Social influence	.42	.06	0.30 to 0.54	.39	<.001
	Performance expectancy	.31	.06	0.19 to 0.43	.31	<.001
	Effort expectancy	.20	.06	.0.09 to 0.31	.22	<.001
	Facilitating conditions	.06	.05	−0.03 to 0.16	.07	.19

^a^General self-efficacy short form (ASKU) [61].

^b^Patient Health Questionnaire-4 (PHQ-4) [65].

^c^Subjective prognosis of work ability scale (SPE) [66].

^d^eHealth literacy scale (eHEALS) [63].

### Secondary Outcome

Overall, eHealth and IT literacy, knowledge of eHealth interventions, and self-efficacy were moderate. Mental distress ranged from “normal” (CAR) to “moderate” (PSY). Risk of incapacity to work was on average. Acceptance did not differ in extreme-group comparisons (PHQ-9: *t*_285_=0.54, *P*=.59; SPE: *t*_214_=1.48, *P*=.14). Participants reported low health-related Internet or mobile use, with less than 1 in 10 participants who indicated utilization for mental health problems or occupational stress. Significant differences of diagnostic groups were observed in all secondary outcome variables. Secondary outcome is reported in Table 4. Post-hoc tests revealed a distinguished role of the pediatric (higher IT literacy, eHealth literacy, eHealth knowledge, eHealth experience, lower ratings on stress through permanent availability, and Internet anxiety) and the psychosomatic subsample (low eHealth literacy, eHealth knowledge, eHealth experience, low self-efficacy, and high Internet anxiety). When including age as a covariate, these group differences remained significant.

**Table 4:** Means, standard deviations, and test statistics of group differences for secondary outcome measures (N=338).

Variable	PSY^a^	OPE^b^	CAR^c^	PED^d^	SUD^e^	Total	*F*	*P* value
	Mean (SD)	Mean (SD)	Mean (SD)	Mean (SD)	Mean (SD)	Mean (SD)		
Performance expectancy	2.41 (1.21)	2.26 (1.09)	2.59 (1.07)	3.24 (1.14)	2.34 (1.06)	2.54 (1.17)	*F*_4,333_=6.23	*P*<.001
Effort expectancy	2.70 (1.29)	2.60 (1.13)	2.98 (1.16)	3.42 (1.03)	2.57 (1.20)	2.83 (1.22)	Welch *F*_4,134.92_=5.64	*P*<.001
Social influence	2.52 (1.06)	2.41 (1.14)	2.79 (0.96)	2.87 (0.97)	2.38 (1.10)	2.59 (1.05)	*F*_4,333_=2.57	.04
Facilitating conditions	3.09 (1.38)	2.95 (1.30)	3.30 (1.19)	3.98 (1.19)	2.91 (1.24)	3.22 (1.32)	*F*_4,333_=6.10	*P*<.001
eHealth literacy^f^	3.30 (1.00)	3.63 (0.90)	3.76 (0.90)	4.08 (0.73)	3.37 (1.13)	3.56 (1.00)	Welch *F*_4,134.52_=9.37	*P*<.001
Internet anxiety	2.57 (1.27)	2.28 (1.14)	2.10 (1.28)	1.87 (1.06)	2.24 (1.08)	2.28 (1.22)	*F*_4,333_=3.76	*P*<.001
Self-efficacy^g^	3.10 (0.91)	4.07 (0.87)	4.11 (0.71)	3.50 (0.99)	3.67 (0.72)	3.57 (0.94)	Welch *F*_4,132.09_=20.75	*P*<.001
Knowledge of eHealth interventions	2.43 (1.10)	3.08 (1.03)	2.90 (1.03)	3.28 (1.05)	2.73 (1.15)	2.78 (1.12)	*F*_4,333_=6.99	*P*<.001
Health-related Internet and mobile use	1.38 (0.55)	1.51 (0.65)	1.71 (0.77)	1.97 (0.86)	1.55 (0.77)	1.58 (0.73)	Welch *F*_4,124.91_=6.28	*P*<.001
Permanent availability stress	3.07 (1.37)	3.03 (1.26)	2.81 (1.31)	1.77 (1.08)	2.72 (1.37)	2.76 (1.37)	*F*_4,333_=9.55	*P*<.001
Mental distress^h^	7.34 (3.21)	3.36 (3.24)	1.84 (2.29)	--	3.21 (2.85)	4.69 (3.77)	*F*_3,283_=61.72	*P*<.001
Risk of incapacity to work^i^	1.41 (1.14)	1.38 (0.95)	0.89 (1.12)	--	1.07 (1.10)	1.22 (1.12)	*F*_3,278_=3.79	.01
IT literacy	2.88 (1.19)	3.02 (1.14)	3.24 (1.18)	4.1 (0.76)	2.93 (1.25)	3.16 (1.2)	Welch *F*_4,134.68_=19.56	*P*<.001

^a^PSY: psychosomatic medicine and psycho-oncology.

^b^OPE: orthopedics.

^c^CAR: cardiology.

^d^PED: pediatric disorders of adolescent patients.

^e^SUD: substance use disorders.

^f^eHealth literacy scale (eHEALS) [63].

^g^General self-efficacy short form (ASKU) [61]

^h^Patient Health Questionnaire-4 (PHQ-4) [65].

^i^Subjective prognosis of work ability scale (SPE) [66].

Furthermore, the majority of inpatients with valid answers (n=280) indicated interest in digital health-related information (68.9%, 193/280) and exercises (56.8%, 159/280). Half of the patients were interested in a Web-based expert contact (52.1%, 146/280) and the use of an app for aftercare (37.9%, 106/280). However, most participants with valid answers preferred face-to-face aftercare (53.4%, 179/335) over Web-based aftercare (4.8%, 16/335), 38.2% (128/335) rated Web-based aftercare as an add-on to traditional methods and 3.6% (12/335) had no interest in any form of aftercare (*χ*^2^_3_=247.99, *P*<.001).

Inter-rater reliability of open answers on advantages and disadvantages was high (Cohen kappa=.89, *P*<.001, 95% CI 0.54-1). Flexibility in terms of time and location of utilization were rated as main advantages, while impersonality and concerns about data security were perceived as main disadvantages of Web-based aftercare. Table 5 contains the most frequently reported advantages of 113 and disadvantages of 121 participants.

**Table 5:** Advantages and disadvantages of Web-based aftercare as measured by number of statements. Frequencies above 5% reported; infrequent statements aggregated in “others” category.

Category		n (%)
**Advantages (n=152 statements)**		
	Flexibility in time	51 (33.6%)
	Local flexibility	33 (21.7%)
	Availability of personal support	11 (7.2%)
	Reduced expenditure of time	9 (5.9%)
	Availability and topicality of health information	8 (5.3%)
	Anonymity	8 (5.3%)
	Other	32 (21.1%)
**Disadvantages (n=142 statements)**		
	Too impersonal	39 (27.5%)
	Concerns about data security	14 (9.9%)
	Increased expenditure of time	14 (9.9%)
	Organizational conflicts	12 (8.5%)
	Insufficient professional supervision	10 (7.0%)
	Insufficient knowledge of Internet use	10 (7.0%)
	General concerns about Internet use	8 (5.6%)
	Insufficient motivation	8 (5.6%)
	Other	27 (19.0%)

## Discussion

### Principal Findings

This study examined barriers and motivators to acceptance of Web-based aftercare in a transdiagnostic sample of inpatients building upon the UTAUT [23] and explored subgroup-specific effects.

The results indicated a rather low level of acceptance of Web-based aftercare for work-related stress. Taken together, 81.6% of the patients reported a low-to-moderate acceptance, which was comparable to previous findings in different clinical settings, such as inpatient diabetic [38], chronic pain [39], or primary care patients [40], and evidence from surveys in the general population [26,27]. Interestingly, acceptance ratings for Web-based aftercare were lower compared to uptake rates of face-to-face-aftercare [7,9]. This was confirmed by a clear face-to-face treatment preference of inpatients in this study, which corresponded to evidence from previous studies [26,27,72]. Despite growing evidence on the efficacy of eHealth interventions [1,2], acceptance in target groups seems limited, which points to different drivers and barriers to eHealth acceptance and adoption. However, high acceptance, eHealth literacy, and Internet-orientation in younger patients suggest sufficient resources to eHealth implementation in adolescent care. Since the incidence rates of mental disorders in adolescents are quite high [73], eHealth interventions thus seem promising in preventing or treating mental health problems.

This study supported the viability of the UTAUT model in assessing acceptance and found that social influence, performance, and effort expectancy, as well as stress through permanent availability, predicted acceptance. These factors may provide a framework for improving acceptance and implementation of eHealth interventions in inpatient rehabilitation.

The effect of *social influence* on acceptance was prominent, in contrast to some previous evidence, where this predictor reached smaller effects [48]. The influence of significant others in eHealth acceptance (family, friends, general practitioner) thus underlines the systemic aspect of the person-environment interaction in clinical settings. A positive attitude of significant others about eHealth efficacy and practitioners’ willingness to refer these programs can foster adoption and adherence in patients. In particular, general practitioners may play an important role as gate keepers to eHealth adoption [74,75]. A study by van Voorhees et al [75] could demonstrate, for example, that uptake of a e-mental health intervention increased with client-centered information by clinicians, targeting intrinsic motivation. Therefore, facilitation of acceptance needs to include relevant stakeholders of health care (eg, practitioners, clinicians, administrators) as important mediators of eHealth implementation.

In line with previous research [47,48], we identified *performance expectancy* as a key predictor of acceptance, with negative outcome expectations predicting lower intention of utilization. Similarly, research suggests that performance expectancy may also be a predictor of treatment outcome in psychotherapy [76]. However, the disparity between low performance expectancy and actual efficacy of eHealth interventions [2] supports the need for further, transparent eHealth education, targeting common misconceptions, for example, about inferior efficacy in contrast to traditional therapies [26,27,72].

The influence of *effort expectancy* reflects the importance of a fit of usability and adoption in everyday life. This corroborates prior research, suggesting that incongruity of organization and scheduling of aftercare sessions with working life are key aspects of participation rate [7,11,35]. It seems that mechanisms of uptake are, to some extent, comparable between Web-based and face-to-face treatment. However, technical barriers and concerns about data security or impersonality as well as facilitators such as flexibility or anonymity are unique to eHealth interventions. Usability may relate to eHealth literacy as well [77], which was above average in this study and comparable to previous research in a student population [63]. However, future studies should expand the concept of eHealth literacy by investigating the ability to utilize eHealth interventions.

Interestingly, *stress through permanent availability* was associated with a lower acceptance, although the effect was very small. It would seem that Web-based aftercare may raise concerns about privacy or technological weariness in coping with health problems. In a qualitative study, Donkin and Glozier [78] identified technology fatigue as an important barrier to adherence to e-mental health interventions. A German survey of 538 employees found that 88% stated being reachable to clients or coworkers, and that 42% could not recognize a clear boundary between professional and private life [79]. Moreover, Reinecke et al [59] found that digital communication load was associated with burnout, depression, and anxiety in a representative survey. However, further studies are recommended to develop a full picture of the association between eHealth utilization, experience of stress through availability, and other sources of occupational stress.

Furthermore, our data show a differential influence of *demographics*, with a pattern of higher acceptance in younger, more educated, and eHealth literate patients with private Internet access and those with eHealth experience. This pattern confirms previous evidence on a relationship with Internet use [80], Internet competence [81], and acceptance of e-mental health interventions [27]. It may be that these mediate the influence of Internet affinity and prior use of eHealth interventions on acceptance. However, we could not find a continuous relation of acceptance and age. Nevertheless, our data point to a “digital divide” in eHealth adoption that may reflect existing social barriers to education or health promotion [82,83]. A recent survey, for example, revealed that only 30% of Germans older than 60 years used the Internet on a regular basis [80]. In this regard, Renahy et al [83] criticized limited access to eHealth not only in old aged but also in chronically ill patients. Future research should therefore aim to facilitate access and education and to adapt eHealth interventions to undersupplied or risk groups in particular.

However, other predictors did not prove to be relevant barriers or resources to eHealth acceptance in our sample. It would seem that a technical infrastructure is widely available and accessible to the majority of inpatients. As previously noted, attitudes may have a greater impact compared with structural barriers on utilization and adherence of eHealth interventions, in particular with ongoing technical progress [25]. However, eHealth intervention knowledge, eHealth experience, and health-related Internet and mobile use were low in most inpatients in our study. This can be applied to health professionals too, where previous studies found that only few practitioners offer e-mental health interventions to patients [84,85], thus limiting availability and exposure to effective treatment tools. Perle et al [52] found that 75% of clinical psychologists would reconsider using a Web camera–based e-mental health intervention after appropriate education. However, eHealth training for health professionals is scarce. When considering methods to facilitate acceptance, initial evidence in different target groups suggests that a brief video-based introduction or interactive presentation containing information about effectiveness, data security, or advantages of eHealth interventions can be an economic way to facilitate acceptance and resources to eHealth adoption [38-40,86]. Recently, Donovan et al [87] provided promising, yet mixed, evidence for a PowerPoint-based eHealth education in mental health professionals. However, further work is required to establish the viability of eHealth training and acceptance facilitation in different clinical contexts such as inpatient rehabilitation and to enhance familiarity and experience with eHealth interventions.

### Limitations

Despite the strengths of our study, several limitations should be considered when interpreting the results. First, since we adapted items to the specific context of our study, psychometric validity cannot be fully ensured. To maintain parsimony, some predictors were operationalized with only 2 items, limiting their reliability. Also, differences in diagnostic groups may partly reflect diverging areas of application of Web-based aftercare between the adolescent and the adult populations. Future designs should increase item quantity to improve reliability of measurement and perform confirmatory factor or moderator analyses with larger sample sizes.

Second, the generalizability to other samples might be limited due to possible selection effects and diverging recruitment efficacy. This is reflected in a low overall response rate, ranging from 26% to 100% between facilities. In a way, the response rate may also represent an indicator of low acceptance of eHealth interventions. Furthermore, a large proportion of adult participants (36.4%) were not working before treatment, which might have limited acceptance of work-related interventions. Moreover, small sample sizes of some subgroup analyses (eg, diagnostic group comparisons, open answers) need to be considered. However, we were able to investigate a broad range of the most common diagnostic groups of German inpatient rehabilitation [88] without preselection of Internet-oriented individuals. Our study relied on a well-established theoretical model and validated secondary outcome measures and proved their reliability in, and applicability to, a mixed inpatient sample. However, future research should promote reliable instruments of acceptance evaluation in different contexts. The aforementioned APOI [37] found the factors “Scepticism and Perception of Risks,” “Confidence in Effectiveness” (similar to performance expectancy), “Technologization Threat” (similar to stress through constant availability), and “Anonymity Benefits” (p. 140) as dimensions of attitudes toward e-mental-health interventions. Moreover, Boß et al [89] recently adapted the Client Satisfaction Questionnaire [90] to eHealth interventions. Also, Mohr et al [91] developed a 24-item questionnaire measuring perceived barriers to psychological treatment, which could be adapted to the context of eHealth interventions. Prospectively, these could also provide clinicians with reliable and valuable information in the implementation of eHealth interventions in practice.

Third, it should be noted that in line with previous studies, we measured acceptance as behavioral intention, which allows only for a prediction but not for direct translation into actual use behavior, given the well-known intention-behavior gap [92]. Future research should therefore include uptake rate as an outcome measure to improve interpretability of motivational aspects and allow for a direct comparison of utilization of Web-based and face-to-face aftercare. Fourth, not only motivational but also structural barriers to eHealth adoption need to be considered when investigating acceptance. Institutional or technical constraints may limit application of eHealth interventions [93]. In Germany and other countries, the use of eHealth interventions still underlies legal constraints, requiring at least an initial face-to-face contact with practitioners. Thus, even if willingness to use eHealth interventions was higher, uptake could be limited by such macro-level constraints.

Finally, the cross-sectional design of the study does not allow for a direct projection to further technical developments and progress of implementation of eHealth interventions. Longitudinal designs will be needed to accompany technical progress and change in underlying barriers and facilitators to acceptance.

### Conclusions

Our study contributes to the exploration of barriers and drivers to eHealth implementation in inpatient treatment and illustrates a limited but developable acceptance. Our results suggest that expectations on efficacy and usability, social norms, and experience of permanent availability modulate acceptance of Web-based aftercare. Thus, it seems critical not only to increase eHealth experience and literacy but to facilitate positive attitudes and target misconceptions about eHealth interventions, regarding competitiveness to face-to-face treatment, effectiveness, individuality, or therapeutic relationship. More elaborate forms of education and quality assurance in implementation are needed, for example, with the development of mandatory and comprehensible quality criteria or training in the application of eHealth interventions for health professionals. Future developments of eHealth interventions therefore should include patients as well as other stakeholders in a collaborative approach to allow for a better conjunction between technical, contextual, and motivational factors influencing adoption and effectiveness of eHealth interventions.

## Appendix

Multimedia Appendix 1 Exemplary description of different Web-based aftercare interventions following inpatient treatment.

Multimedia Appendix 2 Characteristics of the sample.

Multimedia Appendix 3 Correlation matrix of acceptance predictors.

## Abbreviations

ASKU: general self-efficacy short form
CAR: cardiology
CBT: cognitive behavioral therapy
eHEALS: eHealth literacy scale
OPE: orthopedics
PED: pediatric disorders of adolescent patients
PSY: psychosomatic medicine and psycho-oncology
PHQ-4: Patient Health Questionnaire-4
SPE: subjective prognosis of work ability scale
SUD: substance use disorders
UTAUT: unified theory of acceptance and use of technology
